# Appy Christmas

**DOI:** 10.1186/2041-2223-2-25

**Published:** 2011-12-01

**Authors:** Mark A Jobling

**Affiliations:** 1 Department of Genetics, University of Leicester, University Road, Leicester LE1 7RH, UK

## 

What to write, what to write? Christmas is a-coming, so perhaps a seasonal item would be appropriate. But a truly Christmas-focused genetical piece would be a tough task - there's Christmas disease (haemophilia B), of course, but that has nothing to do with the season, taking its name from Stephen Christmas, its first recognized sufferer. And PubMed provides no new inspiration, only an enveloping sense of anti-festive dread from the biomedical literature: '*A Christmas tree in the larynx*' [1] tells the alarming tale of a 2-year-old boy who had a spiky plastic decoration lodged in his throat for several months; '*Christmas lights in the gastrointestinal tract' *[2] sounds even worse, but thankfully is a radiographer's metaphor; '*Christmas-related eye injuries: a prospective study*' [3] warns of the ocular dangers of actual Christmas trees; '*Asthma induced by latex from 'Christmas flower*'' [4] recasts your cheerful poinsettia as a potentially lethal assailant; the threat is extended to yet another species and to another holiday period by '*Immediate allergic and nonallergic reactions to Christmas and Easter cacti' *[5]; and if any of these ills should befall you, remember that it's a bad time to be admitted to hospital (see '*Christmas and New Year as risk factors for death*' [6]). Indeed, you may be lucky to get a bed at all - '*Deck the halls with rows of trolleys*' [7], as one article's title gleefully puts it.

In a few weeks' time, many people will be hoping for a product in their stocking from the world's most valuable company, Apple. This text is being written on an iMac, an iPad sits adjacent, happily charging via its umbilical cord, and there's an iPhone in my pocket. Even the traditional advent calendar has been supplanted by an electronic impostor from the App Store. The 'I'm a PC' brigade may be groaning at this, but let's face it, such devotion to Apple products is hardly atypical these days. At least I started early, when Apple was more cult than craze, writing my PhD thesis long ago on a Mac Plus with its 9-inch monochrome screen. It needed an external hard drive, with a mighty fan to cool its massive 20 megabytes.

The early impetus came from John Edwards (1928-2007), Professor of Genetics at the University of Oxford and describer of the eponymous syndrome (trisomy 18), who was already a Mac enthusiast when I arrived there as a graduate student in 1986. John was the quintessential absent-minded professor (one day arriving at work wearing two ties), his office piled high with papers and strange artefacts. But he was in many ways ahead of his time, combining expertise in clinical and population genetics with skills in statistics and computing. Although he was often difficult to understand, he had a way with an analogy, once describing the panoply of human germline mutation by declaring that 'every ejaculate contains a copy of McKusick's book* [8]'.

John Edwards' lectures on human genetics, which I attended as an undergraduate, were baffling yet strangely memorable. Back then there was no sign-in sheet for lectures, so students came if they felt like it. At the beginning of the course attendance was good, but it rapidly faded under assault from a mysterious collection of slides showing a tent, Greek vases, playing cards, etc. This was pre-Powerpoint, and there was no projected text whatsoever to interrupt the flow of seemingly random images that accompanied the enthusiastic but eccentric lecture. Another exciting stochastic element was provided at the start by the threat of an inappropriate button-press causing the tray of 35mm slides to move backwards, rather than forwards, precipitating its contents onto the floor.

35mm slides have now gone, and Powerpoint (and, to a lesser extent, its Apple relative, Keynote) has become a ubiquitous accompaniment to scientific talks, and a safety net for the speaker. Serried ranks of hierarchical bullet-points act as a 'festival of bureaucratic hyper-rationalism', as vehement critic Edward Tufte has put it [9], and the comforting inevitability of the slideware advancing through the talk leads the argument.

So universal has the comfort blanket of the Powerpoint presentation become, that it's a bit scary to stand in front of an audience without one. Presenting to Café Scientifique (http://www.cafescientifique.org) is a way to feel the fear: one stipulation of taking part in one of these public events is that no slides are allowed, just a ~20-minute talk in a bar or café venue followed by a drink, then an hour or two of questions and discussion with a public audience. Speaking on an evolutionary topic, as I recently did in Glasgow, carries the additional frisson that the audience might include strongly religious types who don't, shall we say, take a conventional scientific view on some aspects of biology.

Luckily for me, this didn't happen, although we did at least get on to the subject of virgin birth. This phenomenon ('parthenogenesis', in its Greek translation) is not uncommon among insects, fish, reptiles and amphibians. Some all-female species, such as the Desert Grassland Whiptail lizard (*Aspidoscelis uniparens*) produce offspring without the intervention of a male, although the process is helped along by mating rituals and 'pseudocopulation'. No such fun for a female zebra shark (*Stegostoma fasciatum*) at the Burj Al Arab aquarium in the UAE [10]: the species is normally sexually reproducing, but this individual produced viable young without any help at all - a creative solution to loneliness, perhaps.

From a selfish point of view, asexual proceedings make sense because there is maximum genetic pay-off for the investment of resources by the mother. But the price is genetic uniformity, and easy hunting for parasites and pathogens: this explains why sex, the route to diversity, is almost universal. Asexual species are often transient, or can switch to the sexual mode: the most impressive exceptions are the bdelloid rotifers, microscopic and near-microscopic animals living in fresh water and damp soil. This group contains over 450 all-female species that have survived without sex for tens of millions of years. They have evolved some tricks that help them - to gain diversity, promiscuously incorporating the DNA of plants, fungi and bacteria in their genomes [11]; and, to escape parasites, simply drying up and blowing away on the wind to colonize healthier environments [12]. In humans and other mammals, parthenogenesis is not a route to viable offspring, because paternally imprinted genes are required. A child whose blood DNA matched just one copy of the maternal genome [13], indicating a parthenogenetic origin in a maternal oocyte, was nevertheless male, giving away the presence of paternal genetic material that permitted development and survival as a chimaera.

Such virgin birth ruminations were the closest my own Café Scientifique contribution got to a Christmas theme. December's talk is appropriately more seasonal, exploring the science behind a popular and energetic non-Apple Christmas gift, the Nintendo Wii. Have fun - and mind that tree!

* The hard-copy forerunner to OMIM (http://www.omim.org)
